# Innovative application of a traffic-prediction spatio-temporal graph convolutional network for dengue disease forecasting

**DOI:** 10.1038/s41598-026-36225-7

**Published:** 2026-01-17

**Authors:** Negar Siabi, Rackhun Son, Maik Thomas, Christopher Irrgang, Jan Saynisch-Wagner

**Affiliations:** 1 Earth System Modelling, GFZ Helmholtz Centre for Geosciences, Potsdam, Brandenburg Germany; 2 Department of Environmental Atmospheric Sciences, Pukyong National University, Busan, South Korea; 3 Centre for Artificial Intelligence in Public Health Research, Robert Koch Institute, Berlin, Germany; 4 Department of Earth Sciences, Institute of Meteorology, Free University Berlin, Berlin, Germany

**Keywords:** Dengue, Machine learning, Climate change, Socio-economic factors, Forecast, Computational biology and bioinformatics, Diseases, Health care, Mathematics and computing

## Abstract

Dengue fever, a vector-borne disease, is a major public health challenge. Accurate prediction methods that can better reflect the complexity of the outbreak are essential for dengue prediction and vector control. In this study, we introduce an adapted Spatio-Temporal Graph Convolutional Network (STGCN), originally developed for traffic forecasting, to predict weekly dengue cases in nine countries in South and Central America from 2014 to 2022. In this approach, we use environmental and socio-economic data in addition to climate data and historical dengue case information to capture complex transmission dynamics. We evaluate the STGCN against a Random Forest (RF) model using the same predictors. The evaluation results show that the STGCN model effectively captures outbreak dynamics and short-term trends. This was especially evident in cases where early transmission patterns are critical. In most of the countries analyzed, STGCN outperformed the baseline random forest model, especially in short-term forecasts, and achieved lower forecast errors in most settings. Forecasting performance varied across regions, with $\hbox {R}^2$ values ranging from 0.78 to 0.98 and RRMSE between 0.14 and 0.43 in short-term forecasts. The strength of the STGCN algorithm lies in its ability to capture spatio-temporal dependencies and handle heterogeneous data sources. This has been particularly valuable in areas with a high dengue burden. Although performance of the model varied slightly across countries, our overall findings highlight the robustness and adaptability of STGCN as a graph-based deep learning framework for dengue surveillance and early detection of its outbreaks.

## Introduction

Dengue fever has become a major public health concern, exacerbated by ongoing climate change. Dengue is caused by infection with one of four dengue virus serotypes (DENV-1 to DENV-4), primarily transmitted through the bite of infected Aedes mosquitoes. According to the World Health Organization (WHO), as of April 2024, more than 7.6 million dengue cases, including over 3000 deaths and over 16,000 severe cases, were reported globally (https://www.who.int/emergencies/diseaseoutbreaknews/item/2024-DON518). This represents nearly a threefold increase compared to the same period in 2023, highlighting an accelerating global crisis. As of late 2023, WHO classified dengue as posing a high global risk, emphasizing the urgent need for accurate forecasting of dengue outbreaks and identification of high-risk regions to implement targeted intervention and control strategies.

Dengue transmission is governed by a complex interplay of climatic, environmental, and socio-demographic factors, as well as biological interactions among the virus (DENV), the mosquito vector, and the human host^1–4^. Climatic variables such as temperature, rainfall, and humidity directly affect mosquito breeding cycles and viral replication rates, thereby shaping seasonal transmission patterns^3^. The density of vegetation and specific microclimatic conditions additionally affect the prevalence of different mosquito species and the presence of viruses, particularly in urban areas where modified ecosystems facilitate the coexistence of various Aedes species^5^. Patterns of land use and human mobility serve as vital factors in the spread of dengue, with travel patterns and urban designs allowing for quick viral transmission across neighborhoods and cities^6^. Urban expansion, infrastructure inequalities, and migration dynamics modulate dengue exposure risk in newly urbanized zones, amplifying transmission especially where piped water and sanitation are inadequate^7^. Furthermore, spatio-temporal modeling has confirmed that human mobility acts as a dynamic force shaping urban epidemics, with mobility shifts altering outbreak trajectories at fine geographic scales^8^. Given the multifaceted nature of dengue transmission, it is imperative to employ modeling frameworks capable of capturing the intricate spatio-temporal dynamics and heterogeneous risk factors involved.

Various modeling approaches, including traditional statistical and machine learning techniques, have been applied to forecast dengue outbreaks. Traditional statistical methods such as time-series analyses and regression models^9–11^ offer interpretability but are constrained by assumptions of linear relationships and limited predictor variables^12,13^. To overcome these limitations, machine learning and deep learning models have become increasingly favored due to their capability to capture complex, non-linear disease dynamics. Based on^14,15^, the most commonly used machine learning models are Support Vector Machines (SVM), Random Forest (RF), Neural Networks (NN), and ensemble models. For example,^16^ adopted SVM to identify climate factors predictive of dengue outbreaks. Reference^17^ used RF, regression, and Autoregressive Integrated Moving Average (ARIMA) methods to predict dengue fever counts and outbreaks in three geographic locations. RF was found to be superior to the other algorithms, with 21% and 33% fewer errors compared to Poisson regression and ARIMA, respectively. Reference^18^ evaluated NN and RF classifiers, highlighting their effectiveness in determining the clinical severity of dengue infections, while^19^ developed a model for predicting monthly dengue cases in Brazilian cities. They compared different machine learning algorithms and feature selection methods using epidemiologic and meteorological variables. They showed RF’s superior accuracy in predicting monthly dengue cases relative to NN, SVM and other regression models. Recently, advanced deep learning models such as Long Short-Term Memory (LSTM) networks and Convolutional Neural Networks (CNNs) have been successfully applied to dengue forecasting, as evidenced by their wide adoption across various research fields^20,21^.

For example, Reference^22^ evaluated the effectiveness of a LSTM model versus a RF regression model for predicting dengue incidence in Brazil. Their findings indicated that the LSTM model outperformed the RF model in accurately forecasting future dengue cases across cities of various sizes. Similarly, Reference^23^ employed LSTM to analyze the impact of climate factors on dengue infection and mortality across India. Reference^24^ compared CNN, Transformer, LSTM, and attention-enhanced LSTM (LSTM-ATT) models against traditional machine learning models in Vietnam, concluding that deep-learning-based approaches provided superior performance. Reference^25^ compared various machine learning models, such as RF, SV Regression, and LSTM Networks, for forecasting dengue outbreaks. They further demonstrated that deep learning models outperformed traditional techniques, emphasizing their robustness and accuracy across diverse geographical contexts. The accuracy of dengue prediction models can vary significantly based on the used method, the quality of data, and the specific context of the study^26^. These reviews highlight the importance of developing and evaluating new methods to improve dengue predictions, in order to better understand the complexity of dengue dynamics and enhance forecasting accuracy. To address these challenges, we propose the use of Spatio-Temporal Graph Convolutional Networks (STGCN), a deep learning architecture originally designed for traffic forecasting, as a novel approach for dengue outbreak prediction. We assess its performance alongside an RF model for comparative analysis.

Additionally, despite recent advancements in dengue modeling, Ref.^26^ observed that many studies still predominantly rely on meteorological variables and historical case data, while socio-economic and environmental predictors remain underutilized. In line with this, Refs.^1,2^ emphasize that effective dengue risk mapping requires careful selection of predictors beyond climatic factors, including socio-demographic, environmental, and entomological components to better capture the spatial heterogeneity of transmission risk. Addressing this gap, our study employs a comprehensive dataset comprising 29 predictors, including 14 socioeconomic and 3 environmental variables, evaluated across nine distinct countries in Central and South America. Socioeconomic factors were included not only to capture human vulnerability but also to account for structural inequalities and urban conditions that shape mosquito ecology and disease transmission dynamics^5,7,27^.

## Materials and methods

### Data and study area

**Fig. 1:**
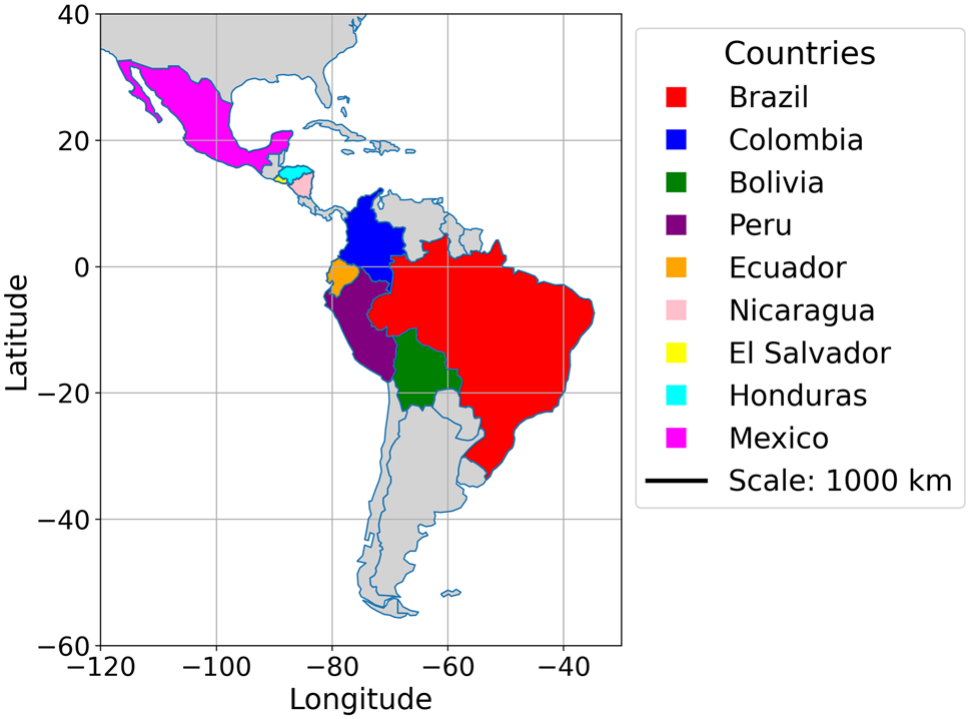
Map showing the study area across Central and South America, highlighting the nine countries analyzed: Brazil, Colombia, Bolivia, Peru, Ecuador, Nicaragua, El Salvador, Honduras, and Mexico. Map generated using Python (GeoPandas 1.0.1 (https://geopandas.org) and Matplotlib 3.8.4 (https://matplotlib.org).

Our study aims to forecast the number of dengue cases on a weekly basis across Central and South America (cf., Fig. 1). We sourced weekly dengue data from Open Dengue Database (https://opendengue.org), which covers 20 countries in our target domain. We specifically analysed data from nine countries, namely Brazil, Colombia, Bolivia, Peru, Ecuador, Nicaragua, El Salvador, Honduras and Mexico, selecting them based on the criterion that missing data should not exceed 10%. To assess the impact of meteorological conditions on dengue transmissions, we incorporated 11 weather factors obtained from NASA (https://science.nasa.gov/earth/data/climate-data). Additionally, we included various socio-economic factors, with data sourced from the World Bank Database (https://data.worldbank.org/indicator). In total, 29 variables were employed as inputs for this study. In general, the applied dataset can be categorized into (1) epidemiologic history, (2) meteorological factors, (3) environmental indicators, and (4) socioeconomic conditions. More details about the data are given in Table 1.

**Table 1:** The information about input variables used for training the models and simulating dengue dynamics. The first column from the left shows the classification of the variables. We used 29 variables (second column), which are grouped into four classes.

Variable name (number of factors)	Description
Dengue case data (1)	Number of dengue cases at the previous week
Meteorological factors (11)	Temperature at 2 Meters ($\circ$°C)
	Dew/Frost Point at 2 m ($^\circ$C)
	Wet Bulb Temperature at 2 m ($^\circ$C)
	Earth Skin Temperature ($^\circ$C)
	Temperature Range at 2 m ($^\circ$C)
	Maximum Temperature at 2 m ($^\circ$C)
	Minimum Temperature at 2 m ($^\circ$C)
	Relative Humidity at 2 m (%)
	Corrected Precipitation (mm/day)
	Wind Speed at 2 m (m/s)
	All Sky Surface Shortwave Downward Irradiance (kW-hr/$\hbox {m}^2$/day)
Environmental factors (3)	Environment-Agricultural land ratio
	Environment-Arable land ratio
	Environment-Forest area ratio
Socio-economic factors (14)	Economy-Gross domestic product (GDP)
	Economy-Gini index
	Health-Immunization DPT vaccine
	Health-Immunization measles rate
	Health-Prevalence of HIV rate
	Infrastructure-Access to electricity ratio
	Infrastructure-Annual freshwater withdrawals
	Infrastructure-Hospital beds
	Population-Ages 0–14 ratio
	Population-Ages 15–64 ratio
	Population-Ages 65 above ratio
	Population-Population in the largest city ratio
	Population-Rural population
	Population-Population density

Epidemiological data included the number of dengue cases in the previous week, capturing recent transmission dynamics. Meteorological factors such as temperature at multiple levels, humidity, wind speed, precipitation, and solar radiation, were selected due to their documented influence on mosquito breeding cycles, viral replication rates, and seasonal transmission peaks^26,28^. Environmental indicators, including agricultural land ratio, arable land ratio, and forest area ratio, reflect the ecological conditions conducive to vector propagation and habitat distribution^29^. Socioeconomic variables spanned domains such as economic performance (e.g., GDP, Gini index), healthcare infrastructure (e.g., hospital beds, immunization coverage, HIV prevalence), access to utilities (e.g., electricity and water withdrawal), and detailed population demographics. These were included based on prior studies demonstrating their influence on outbreak vulnerability, human-vector contact rates, and access to medical care^2,30^.

### Data processing

Since the data came from different sources (Table 1), we classified them based on spatial and temporal resolution. Dengue data is a composite of weekly, monthly and annual data for each country, where we extracted weekly information of dengue cases for each country. We also convert daily weather data to weekly data based on the average of seven days. Then, all data sets were checked for gaps, duplicate data and outliers. For outliers, we used Z-scores and the Grubbs’s test^31^. In the process of data quality review, we found gaps in dengue data and socio-economic data sets. In dealing with missing data, there were variables for certain countries with very large gaps that were excluded during data quality processing. We only applied interpolation to dataset where the number of missing values is less than 10% of the data. After filling the gaps, we reconciled the data in the next step and checked the data again for outliers. To ensure consistency and comparability across predictors, we applied min-max normalization to all input variables prior to model training. While RF does not require normalization, this step was necessary for STGCN, which benefits from scaled inputs.

### Modeling approach

This paper develops and validates a random forest and a spatio-temporal graph convolutional network model, to predict dengue cases. The RF model was chosen as the baseline since previous research has consistently demonstrated its superior performance over regression-based and ARIMA-type approaches in dengue forecasting. The primary objective was to highlight the added value of a graph-based deep learning approach relative to a strong machine learning baseline.

#### Spatio-temporal graph convolutional network (STGCN)

The Spatio-Temporal Graph Convolutional Network (STGCN) is a deep learning architecture originally developed for traffic forecasting^32^, designed to model and predict time series data that concurrently exhibit spatial and temporal dependencies. STGCN employs graph convolution operations to capture complex spatial relationships inherent in graph-structured data, extending traditional convolution operations to accommodate non-Euclidean domains. By representing spatial dependencies using graph structures, the model can effectively identify and learn spatial patterns across interconnected nodes, which is particularly advantageous for irregular data topologies. To capture temporal dependencies, STGCN incorporates gated convolutional layers instead of recursive architectures such as RNNs or LSTMs. This design choice enables parallel computation over temporal sequences, significantly enhancing training efficiency while preserving the model’s ability to capture sequential dynamics. In the context of this study, STGCN is adapted to model the spatial and temporal dynamics underlying dengue transmissions across countries. Each country is represented as a node in a dynamic spatio-temporal graph, where edges encode learnable inter-country relationships that evolve over time. Rather than relying on predefined adjacency matrices derived from geographical proximity or transportation networks, we construct a trainable adjacency matrix with self-loops, where the diagonal elements are initialized to one. This allows the model to autonomously learn optimal connectivity patterns based on the data, thereby capturing latent interaction mechanisms. Although the adjacency matrix is initialized in a trainable form, its learned connectivity patterns are guided by epidemiologically meaningful signals present in the data. Increases in incidence in one region that consistently precede increases in another are captured as stronger edges, implicitly reflecting plausible pathways such as human mobility, trade links, or climatic similarity. This design ensures that the graph structure is not arbitrary, but reflects functional transmission relationships consistent with observed spatiotemporal dynamics. We expect this design is suitable for modelling disease transmission, as the spread of infectious disease is typically influenced by a complex interplay of epidemiological, climatic, demographic and socio-economic factors, many of which are not reflected in fixed and static graphs.

**Fig. 2:**
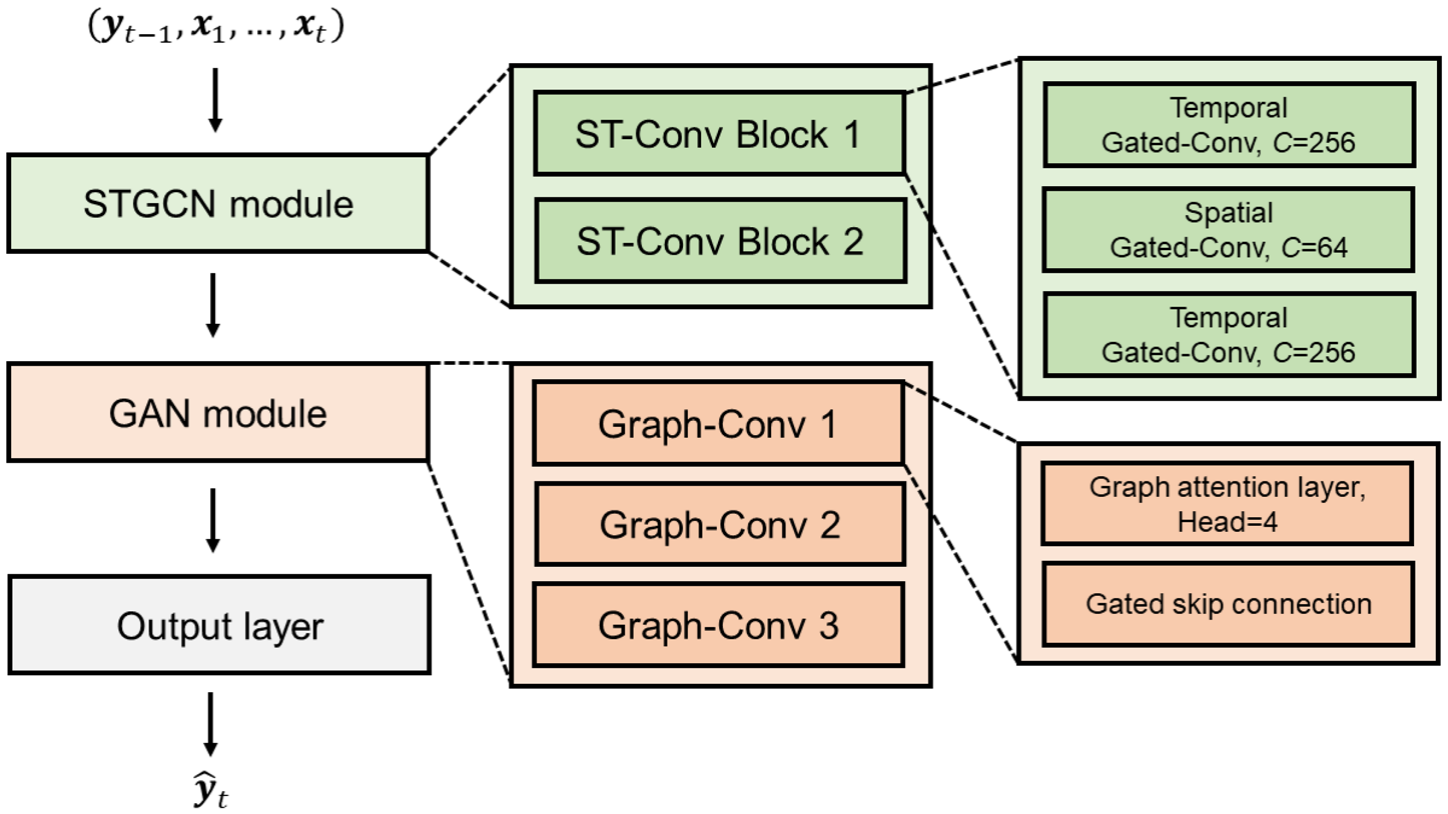
Framework of architectural details of the STGCN model. From left to right, the STGCN framework as the first module integrates the spatial and temporal processing layers into spatio-temporal convolutional blocks (ST-Conv Block 1 and 2), each comprising two temporal gated convolutional (Temporal Gated-Conv) layers with a spatial gated convolutional (Spatial Gated-Conv) layer in between configuring it with hidden dimensions of 256 for temporal layers and 64 for spatial layers. The second module (GAN module) adapts the structure of graph attention network. This module comprises three graphical convolutional layers (Graph-Conv 1,2 and 3), each equipped with gated skip connections and a graph attention mechanism.

The full architecture of the proposed model is illustrated in Fig. 2. The STGCN module consists of two stacked spatio-temporal convolutional blocks. Each block follows a ‘sandwich’ structure, in which a spatial graph convolution layer is inserted between two temporal gated convolutional layers. Temporal convolution layers use one-dimensional causal filters to model sequence dynamics while preserving temporal order, and they permit parallel processing, offering computational advantages over traditional recurrent models. The spatial convolution is based on generalized Chebyshev polynomial approximation^32^, which enables localized spectral filtering of graph-structured inputs. By using this method, the model can efficiently aggregate multi-hop neighbourhood information, while maintaining scalability with respect to graph size. To further enhance spatial feature learning, a Graph Attention Network (GAN) module^33^ is incorporated in parallel with the STGCN pathway. The GAN mechanism introduces self-attention into graph neural networks by computing attention coefficients that reflect the relative importance of neighbouring nodes for each target node. This allows the model to dynamically assign adaptive weights to inter-country influences, capturing heterogeneous interactions that evolve over time. In our implementation, three attention-based graph convolution layers are stacked sequentially, each augmented with gated skip connections to improve gradient flow and stabilize training. The final prediction layer consists of a single fully connected layer that maps the concatenated features to the desired output dimension. Based on preliminary hyperparameter tuning, we set the hidden layer dimension to 256 for the temporal convolutional layers and 64 for the spatial graph convolutional layers. The GAN modules use four attention heads. The model produces weekly dengue case forecasts using a rolling 4-week input window, enabling it to capture ongoing transmission trends and emerging spatial patterns in disease propagation.

#### Random forest (RF)

RF is a supervised machine learning algorithm that utilizes the ensemble learning method^34^. RF is capable of modeling non-linear and complex relationships between features and target variable through the use of multiple decision trees for predictions. This capability is related to the choice of the hyperparameters (number of trees, maximum features, depth etc.). Each tree is trained on a separate subset of the data and usually the results are more accurate than a single tree. Also, the overall performance of the model is less likely to be impacted by noisy data. Distribution of the data at the root level or the correlation between the target variable and features were not considered in random forest due to its non-parametric nature. RF is capable of handling a wide variety of data types including numerical and categorical data without bias since random subsets of features for each decision tree are chosen during training by random forest. Additionally, it can handle high dimensional large datasets. The trees can be created in parallel, which increases the speed of the training stage. One of the most important capabilities of RF is that the importance of each feature can be quantified in this method. In this research, we employed a RF algorithm to predict dengue fever cases. The RF model was implemented with the following parameters: the number of trees in the forest was set to 1000, the maximum depth of each tree was six, and the minimum number of samples required to split an internal node was two. Additionally, we used squared error as the function to measure the quality of a split.

### Training and testing

The dataset for dengue statistics spans from 2014 to 2022 and comprises 470 samples. We utilized data from 2014 to 2020 to train the model, reserving the data from the last two years for model evaluation. This division ensures a robust assessment of generalizability across unseen data. To address the limitation in training data size, we augmented the training dataset by interpolating it from a weekly to a daily timescale. This interpolation effectively increased the number of training samples by a factor of seven. Subsequently, the data were reorganized back into weekly sequences to preserve the original temporal structure. This approach enhances the model’s ability to learn complex temporal patterns without compromising its generalization capability. Temporal interpolation was applied only during the training phase and never during validation or testing. All evaluation metrics were computed on the original weekly series, ensuring that predictive performance reflects skill on true weekly data rather than on artificially densified sequences. The interpolation procedure was designed to regularize training by increasing the number of effective training steps, smoothing abrupt week-to-week jumps into more gradual trajectories, and encouraging the model to learn stable spatiotemporal patterns rather than memorizing coarse snapshots.

To evaluate the impact of temporal interpolation, we conducted a sensitivity analysis comparing model performance with and without data augmentation. The sensitivity analysis confirmed that interpolation improved generalization while preserving validity of cross-validation. Models trained without interpolation showed markedly lower performance in several countries (e.g., Bolivia, El Salvador, Honduras), confirming the regularization benefit of the procedure. Results of this comparison are provided in Supplementary Table S1.

For the STGCN model, we employed 10-fold cross-validation, utilizing ensembles from 10 different trained models to generate the final product. Specifically, we employed a repeated random sub-sampling validation strategy by selecting 10% of the training samples for validation in each iteration. This procedure was repeated 10 times, resulting in 10 models sharing the same architecture but differing in initialization and validation splits. To generate the final prediction, we averaged the outputs of these models, assigning equal weight to each. This ensemble approach enhances prediction robustness and mitigates variance, leading to more stable and reliable model performance. To prevent overfitting, we implemented a dropout rate of 0.5 and a weight decay factor of 1e−6 during optimization.

To solely consider the effect of the factors on the accuracy of forecasts in different geographical regions, we set the same hyperparameters and used the same time series split across different regions. This approach reduces the effect of such factors on the accuracy of the model.

### Model performance evaluation

Our trained models are employed to forecast the test set, and the effectiveness is measured by coefficient of determination ($\hbox {R}^2$), Root Mean Squared Error (RMSE), Nash–Sutcliffe Efficiency (NSE), and Mean Absolute Scaled Error (MASE), as depicted in Equations 1–4. Calculating the coefficient of determination was done to evaluate the level of correspondence achieved when predicting dengue cases. This criterion measures how much there is correlation between the real and predicted data.

The statistical criterion of root mean square error was employed to compare the reported dengue cases for each epidemiological week with the predicted cases. To establish the degree of dispersion between prediction errors, the standard deviation of the residuals is evaluated with the RMSE. The NSE was employed to evaluate how well predicted values replicate the variability of the observed dengue cases. A value close to 1 indicates strong agreement, while negative values suggest poor model performance compared to using the mean. MASE offers a scale-independent error metric, comparing prediction errors with those from a naive forecast based on one-step lagged values. Values below 1 imply that the model predicts better than the naive baseline. Where $n$ is the number of samples, ${\hat{y}}_i$ is the predicted values, $y_i$ is the actual values, ${\bar{y}}_i$ is the average of the actual values, $y_t$ represents the actual (observed) value at time $t$, ${\hat{y}}_t$ is the predicted value at time $t$, and $y_{t-1}$ is the actual value at the previous time step.

1 $$\begin{aligned} R^2 = 1 - \frac{\sum _{i=1}^{n}(y_i - {\hat{y}}_i)^2}{\sum _{i=1}^{n}(y_i - {\bar{y}})^2} \end{aligned}$$

2 $$\begin{aligned} RMSE = \sqrt{\frac{1}{n}\sum _{i=1}^{n}(y_i - {\hat{y}}_i)^2} \end{aligned}$$

3 $$\begin{aligned} \text {NSE} = 1 - \frac{\sum _{i=1}^{n}(y_i - {\hat{y}}_i)^2}{\sum _{i=1}^{n}(y_i - {\bar{y}})^2} \end{aligned}$$

4 $$\begin{aligned} \text {MASE} = \frac{\frac{1}{n} \sum _{t=1}^{n} |y_t - {\hat{y}}_t|}{\frac{1}{n-1} \sum _{t=2}^{n} |y_t - y_{t-1}|} \end{aligned}$$

## Results

The models were trained and evaluated on a dataset of weekly dengue cases from 2014 to 2022. The goal was to better capture the complex spatio-temporal patterns of dengue outbreaks and improve predictive performance by utilizing the STGCN model based on various climate, environmental, and socioeconomic variables. In order to evaluate the performance of the model in time and space, we made weekly forecasts from 1 to 4 weeks in the future. Also, the forecasts were done for several countries in order to measure the performance of the model in different regions.

### Performance of the STGCN model

#### Spatio-temporal performance

**Fig. 3:**
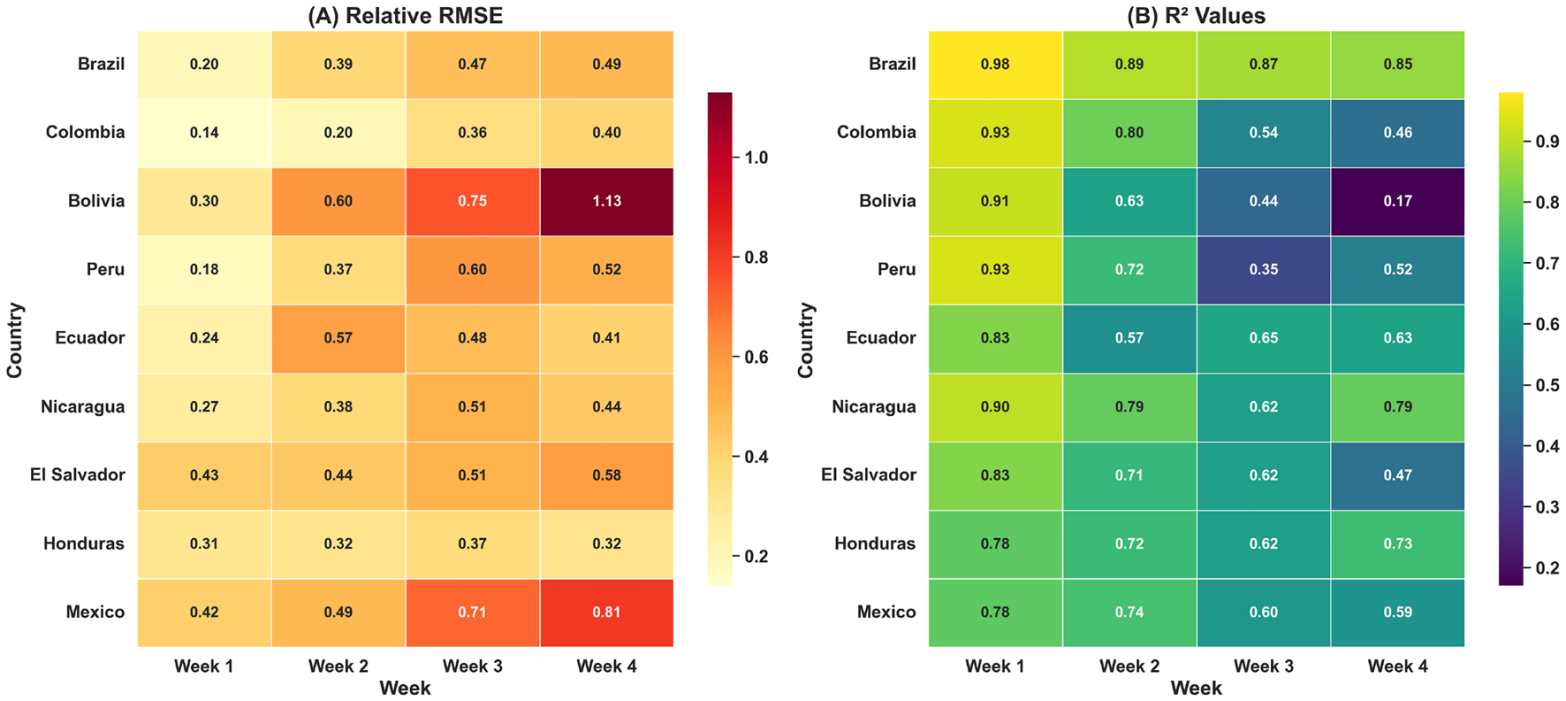
Spatio-temporal performance of the STGCN model in forecasting dengue incidence across nine Latin American countries over four prediction horizons (Week 1 to Week 4). (**A**) Heatmap of Relative RMSE values, where lower values indicate better predictive accuracy. (**B**) Heatmap of $\hbox {R}^2$. values, where higher values reflect stronger model fit.

Figure 3 illustrates the performance of the STGCN model in forecasting dengue cases across nine Latin American countries over four prediction horizons (Week 1 to Week 4). To evaluate model accuracy, two complementary heatmaps are presented: Panel (A) shows Relative RMSE (RRMSE) values, while Panel (B) displays $\hbox {R}^2$ scores. Relative RMSE values are normalized RMSE scores calculated by dividing the RMSE by the mean of observed values. This normalization enables fair comparison across countries with different dengue incidence scales. The RRMSE heatmap reveals that the model performs best in short-term forecasts, particularly in countries such as Brazil, where error values are notably low (e.g., Brazil: 0.20 in Week 1). As the forecast horizon extends, error values increase across most regions, with Bolivia showing the highest error (1.13 in Week 4). This trend is reflected in the $\hbox {R}^2$ heatmap, where values exceed 0.90 in Week 1 for several regions, but decline to 0.17 in longer-term forecasts. Despite the challenges posed by zero-inflated data, the model achieved $\hbox {R}^2$ values up to 0.98 and RRMSE as low as 0.14, indicating strong predictive capability under appropriate conditions. Lower accuracy in some cases reflects inherent data limitations rather than model inadequacy.

#### Temporal performance

**Fig. 4:**
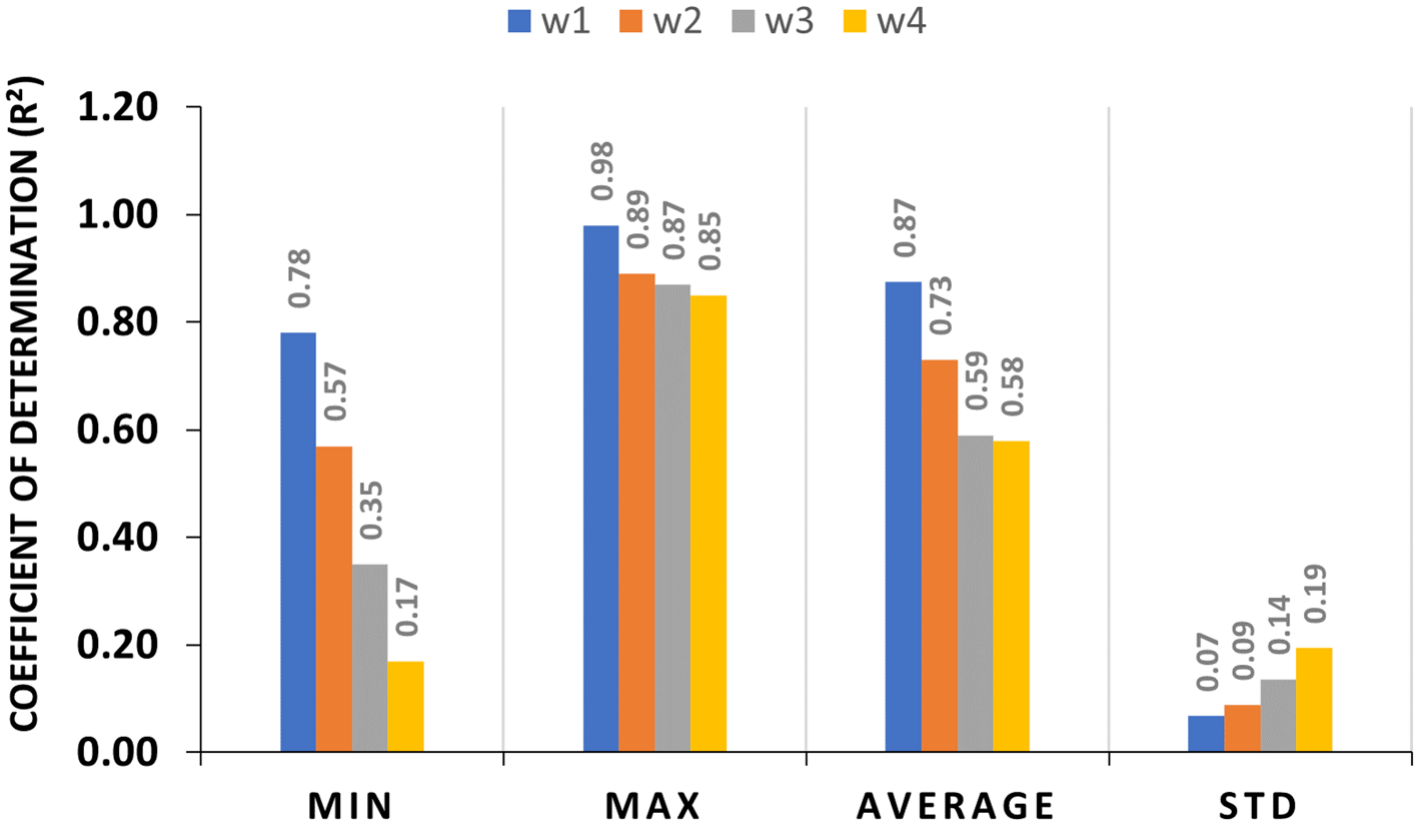
Temporal performance of STGCN models for dengue forecast. The statistics reflect the average $\hbox {R}^2$ values obtained from running the model across all regions for each forecast horizon.

Figure 4 demonstrates the temporal forecasting accuracy of our adopted model (STGCN) in forecasting dengue fever cases across four prediction horizons: 1-week, 2-week, 3-week, and 4-week intervals. The chart illustrates the average $\hbox {R}^2$ values derived from the model’s predictions across all studied regions. According to the results, for week 1 (w1), the accuracy ranges from 0.78 to 0.98, with an average accuracy of 0.87 and a standard deviation (STD) of 0.07 indicating high accuracy of the model in one-week predictions. This consistent performance is reflected in a low standard deviation of 0.07, indicating the reliability and steadiness of our model’s predictions. As the forecast horizon extends, a marginal decline in average accuracy is observed, yet the model continues to deliver stable and reliable predictions.

#### Spatial performance

**Fig. 5:**
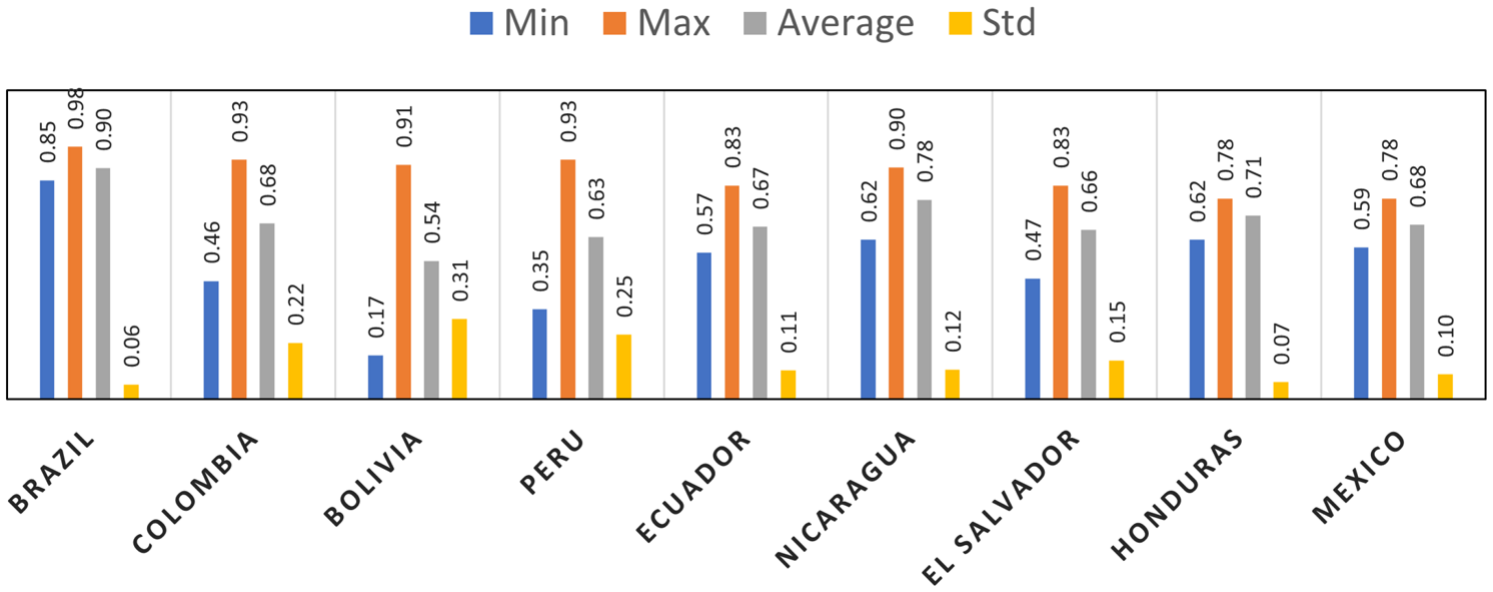
Spatial performance of the STGCN model in forecasting dengue across different regions, measured by $\hbox {R}^2$. The statistics reflect the average $\hbox {R}^2$ values obtained from running the model for each region across all forecast horizons.

Overall performance of the STGCN model in forecasting dengue fever across different regions is shown in Fig. 5. The country with the highest averaged $\hbox {R}^2$ is Brazil, followed by Nicaragua, Honduras and Colombia. The model has the least accuracy in predicting dengue cases in Bolivia, with an average $\hbox {R}^2$ of 0.54 and a standard deviation of 0.31. The averaged $\hbox {R}^2$ values, generally exceeding 0.60, suggest that the STGCN model performs reliably and demonstrates a reasonable level of predictive accuracy. Although the $\hbox {R}^2$ values indicate satisfactory accuracy, further refinement could enhance the model’s performance in certain contexts.

### Comparative performance analysis of STGCN and RF models

**Fig. 6:**
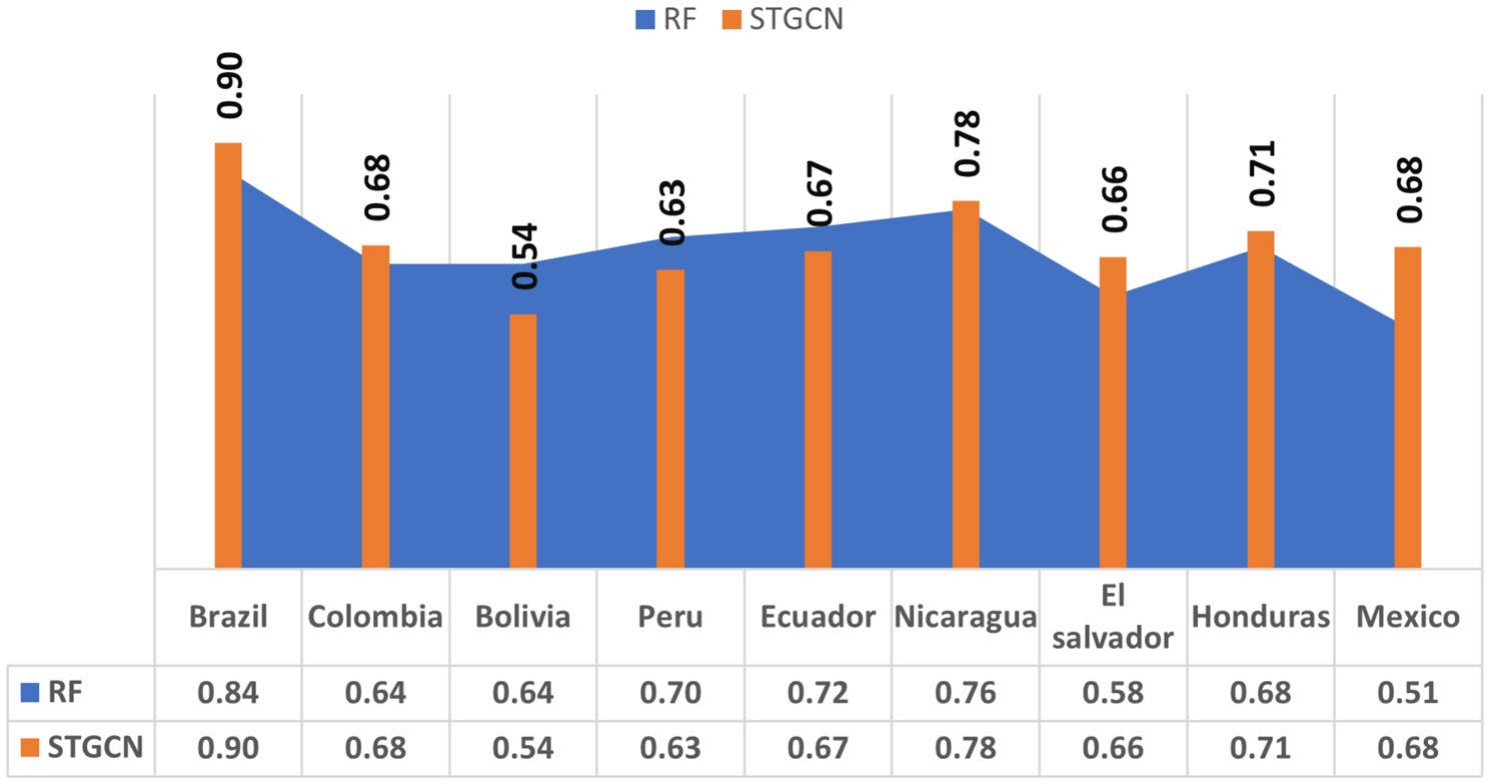
Comparison of STGCN model and RF model overall performance, measured by $\hbox {R}^2$. The statistics reflect the average R-squared values obtained from running the models for each region across all forecast horizons.

For further analysis, we compared the STGCN model with the Random Forest (RF) baseline using multiple validation metrics, including $\hbox {R}^2$, Relative RMSE, Nash–Sutcliffe Efficiency (NSE) and Mean Absolute Scaled Error (MASE). Figure 6 presents the average $\hbox {R}^2$ values for each country across four forecast horizons for both models. As illustrated in the figure, the STGCN model achieved higher average $\hbox {R}^2$ values in Brazil (0.90 vs. 0.84), Colombia (0.68 vs. 0.64), Nicaragua (0.78 vs. 0.76), El Salvador (0.66 vs. 0.58), Honduras (0.71 vs. 0.68), and Mexico (0.68 vs. 0.51), indicating stronger predictive accuracy. However, the RF model outperformed STGCN in Bolivia (0.64 vs. 0.54), Peru (0.70 vs. 0.63), and Ecuador (0.72 vs. 0.67), suggesting context-specific advantages.

**Fig. 7:**
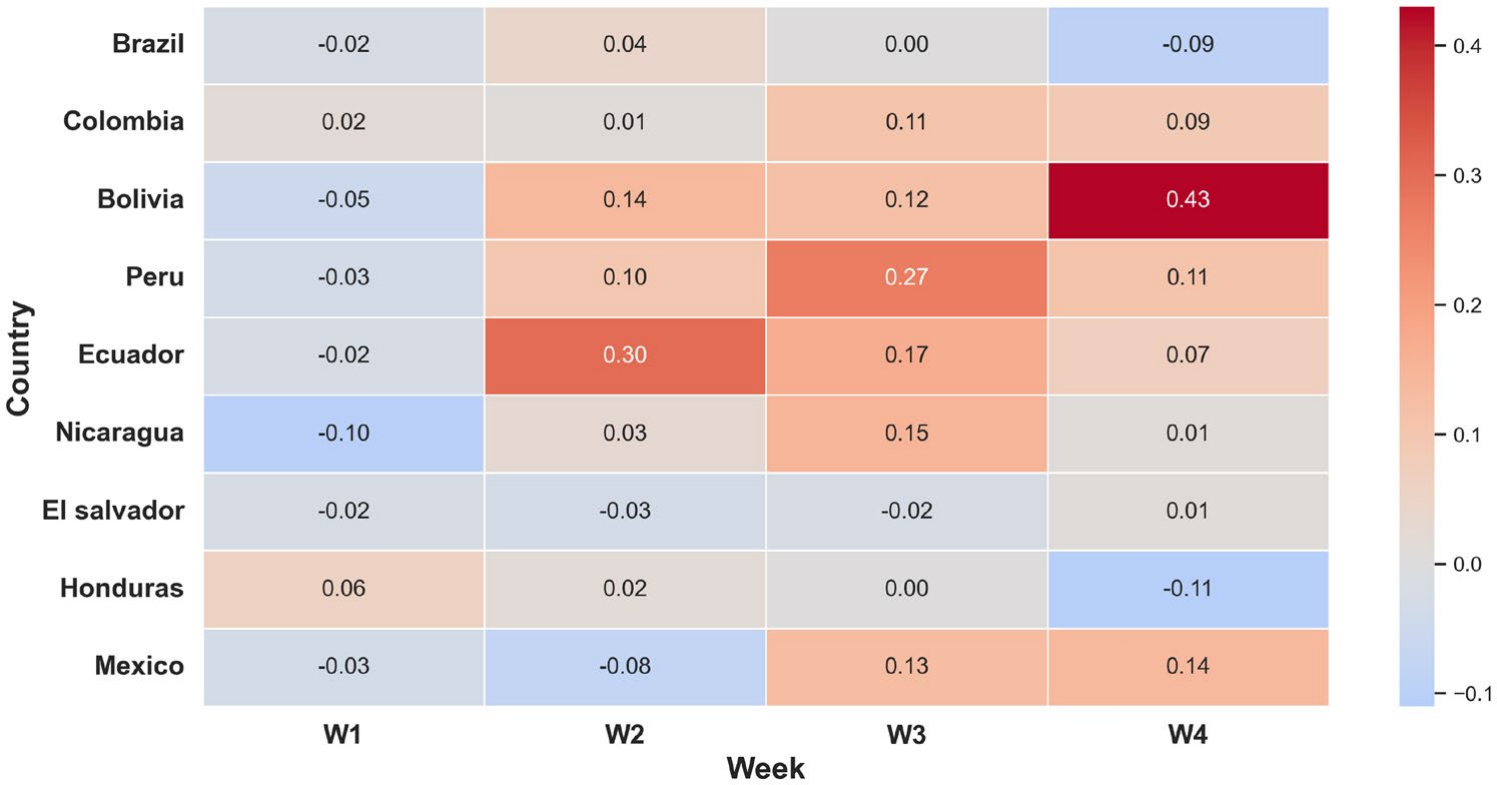
Heatmap of the difference in Relative RMSE between STGCN and RF models across four forecast horizons (Weeks 1–4) for nine Latin American countries. Negative values (blue) indicate superior performance by STGCN, while positive values (red) reflect better results from RF. The color gradient highlights temporal and regional variations in model effectiveness.

Figure 7 presents a heatmap illustrating the difference in Relative RMSE between the STGCN and RF models across four forecast horizons (Weeks 1 to 4) and nine Latin American countries. Negative values indicate that STGCN surpassed RF in predictive accuracy, whereas positive values suggest RF performed better. As illustrated, STGCN consistently records lower RRMSE values during early prediction periods, especially in Week 1, where the majority of countries show negative differences. However, as the forecast period lengthens, the superiority of STGCN decreases. These results suggest that the STGCN model maintains strong predictive accuracy in short-term horizons and demonstrates reasonable spatial generalizability.

To provide a more nuanced view of model performance, in addition to the $\hbox {R}^2$ and Relative RMSE metrics, Table 2 presents a comparison of model performance using Nash–Sutcliffe Efficiency (NSE) and Mean Absolute Scaled Error (MASE). These additional indicators offer broader insight into temporal consistency and comparative accuracy. Among the nine countries assessed, the STGCN model exceeded the RF in six instances, achieving both lower MASE and higher NSE scores. This includes Brazil, Ecuador, Nicaragua, El Salvador, and Mexico, where STGCN consistently exhibited greater predictive accuracy. In two countries (Bolivia and Peru), the outcomes were varied. The STGCN model showed superior performance based on the NSE criterion; however, when assessed using the MASE criterion, a minor, but not significant, difference was noted between the two models, favoring RF. Elevated NSE values reflect stronger alignment with observed dengue patterns, while reduced MASE scores indicate improved reliability in capturing seasonal dynamics. Notably, STGCN maintained its advantage even in regions with sparse or noisy surveillance data, underscoring its generalization capability and robustness to data irregularities.

**Table 2:** Comparison of predictive performance between STGCN and RF models for dengue forecasting across nine countries in short-term. Note: Lower MASE and higher NSE values indicate superior predictive performance.

Country	MASE (RF)	MASE (STGCN)	NSE (RF)	NSE (STGCN)	Model comparison
Brazil	1.52	1.30	0.951	0.969	STGCN better in both metrics
Colombia	1.33	1.36	0.891	0.855	RF better in both metrics
Bolivia	0.443	0.445	0.880	0.902	Mixed: STGCN better in NSE, RF better in MASE
Peru	0.947	0.967	0.896	0.906	Mixed: STGCN better in NSE, RF better in MASE
Ecuador	1.14	0.96	0.792	0.810	STGCN better in both metrics
Nicaragua	1.42	1.09	0.766	0.889	STGCN better in both metrics
El Salvador	1.10	0.94	0.623	0.694	STGCN better in both metrics
Honduras	0.76	0.97	0.822	0.734	RF better in both metrics
Mexico	1.00	0.74	0.566	0.737	STGCN better in both metrics

Overall, the STGCN model demonstrates superior predictive performance, greater temporal stability, and enhanced adaptability across diverse epidemiological settings, positioning it as a robust tool for dengue outbreak forecasting.

### Dengue outbreaks predictions

**Fig. 8:**
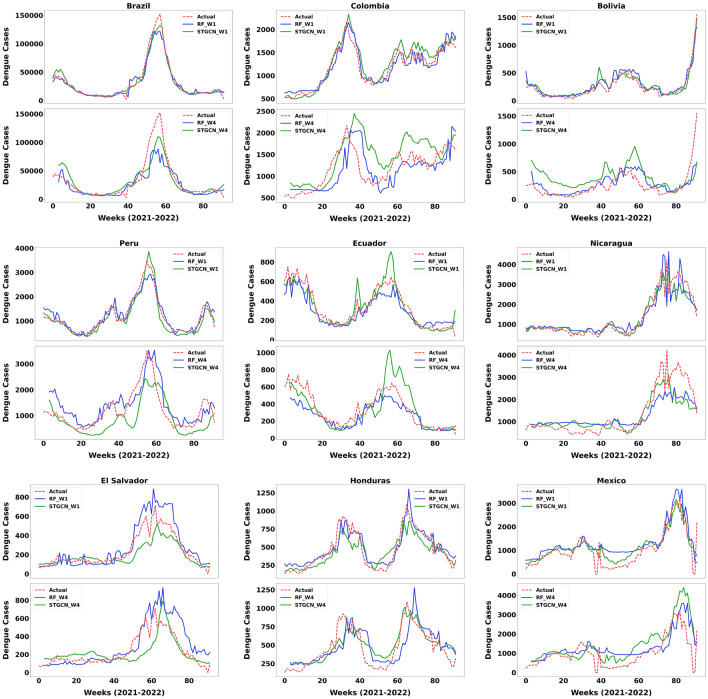
Forecasting dengue outbreaks over one-week and four-weeks forecast horizons across various countries.

Figure 8 presents the actual reported dengue cases and those forecasted by the STGCN and RF models for different regions. In these diagrams, the horizontal axis values are the weeks of the validation years 2021–2022. The vertical axis is the dengue case values per epidemiological week. The upper and lower graphs correspond to the model’s predictions at one week and 4 weeks forecast horizons, respectively. In general, both models are able to predict the pattern and trend in the data well. This can be considered especially in the case of a sudden increase in the number of dengue cases.

To interpret the results appropriately, it is important to clarify how outbreaks were defined in this study. International agencies such as WHO and Pan American Health Organization (PAHO) generally define an outbreak as “an increase in cases above the expected baseline”(https://www.emro.who.int/health-topics/disease-outbreaks). However, there is no universally accepted quantitative threshold for defining outbreaks, as baselines depend on country specific historical data, surveillance systems, and epidemiological context. Since such standardized baselines were not available for this methodological study, we adopted a simplified proxy definition of outbreaks as a sudden increase in the number of dengue cases that were twice the average value of the validation period as a significant outbreak. This uniform threshold served only as an illustrative tool to demonstrate the model’s ability to capture abrupt increases.

Our results show that both models have good performance on predicting dengue outbreaks. The prediction of the model is consistent with the real data in cases where the dengue cases values are lower than the average and the fluctuation of the data is low. Both models perform well in one week forecast of dengue cases. However, the performance of the models tends to decline as the time period extends. Over a 4-week forecast horizon, the models effectively capture the behavior of the real case numbers. Nevertheless, during significant outbreaks, the models may either underestimate or overestimate the cases, depending on the situation. Although the models exhibit a slight inclination to both under and over predict dengue outbreaks within a four-weeks span, the predictions were still sufficiently accurate to anticipate the outbreaks.

In the case of Brazil, which experienced a significant dengue outbreak in 2022, both models successfully predicted the outbreak but slightly underestimated its severity. The STGCN model showed a smaller discrepancy between predicted and actual dengue prevalence across both time steps. In the case of Colombia, where a significant outbreak was observed in 2021, the one-week forecasts from both models performed well, showing only a slight overestimation. However, this overestimation became more pronounced in the four-week forecast horizon, especially in the STGCN model. In the case of Bolivia, both models performed very well in one-week forecasts, especially during the late 2022 outbreak. Over the 4-week forecast horizon, both models tended to overestimate. In the case of Peru, where the number of dengue cases was significantly high at the beginning of 2022, the models successfully predicted the outbreak over a one-week period. The STGCN model slightly overestimated, but the RF model underestimated at the peak. In the case of the four-week forecast horizon, the RF model performed better at predicting the dengue cases outbreak. In the case of Ecuador, both models effectively predicted the two outbreaks observed in early 2021 and 2022. Generally, the RF model tended to slightly underestimate. The STGCN model showed an overestimation during the 2022 outbreak compared to the RF model in the four-week forecast horizon. In the case of Nicaragua, the STGCN model better predicted the data pattern, while the RF model overestimated the dengue outbreak in mid-2022. Both models underpredicted the dengue outbreak over the four-week forecasting horizon compared to the actual values. For El Salvador, the STGCN model better adhered to the dengue pattern over time, whereas the RF model captured the overall pattern but lacked detailed accuracy. Also, the RF model generally overestimated, while the STGCN model tended to underestimate the dengue outbreak in mid-2022 at both forecasting time steps. In the case of Honduras, two outbreaks were observed in 2021 and 2022, respectively. While the models maintained the dengue fluctuation pattern, they underestimated the 2021 outbreak. The RF model overestimated the second outbreak. However, the RF model performed better in one-week forecasts, but the STGCN model was more accurate in 4-week forecasts in 2022. In the case of Mexico, where the outbreak in late 2022 was significant, the STGCN model accurately predicted it over the one-week time step and in this case, it performed better than the RF model, which overestimated. Over the 4-week time frame, both models maintained the pattern of fluctuations but overestimated in predicting the dengue outbreak. The STGCN model, however, achieves better performance compared to the RF model (see the subsection on the comparative performance analysis). This indicates that the use of the STGCN model has substantially improved the accuracy of the forecasting dengue outbreaks.

### Impact of socio-economic factors on dengue predictions accuracy

**Fig. 9:**
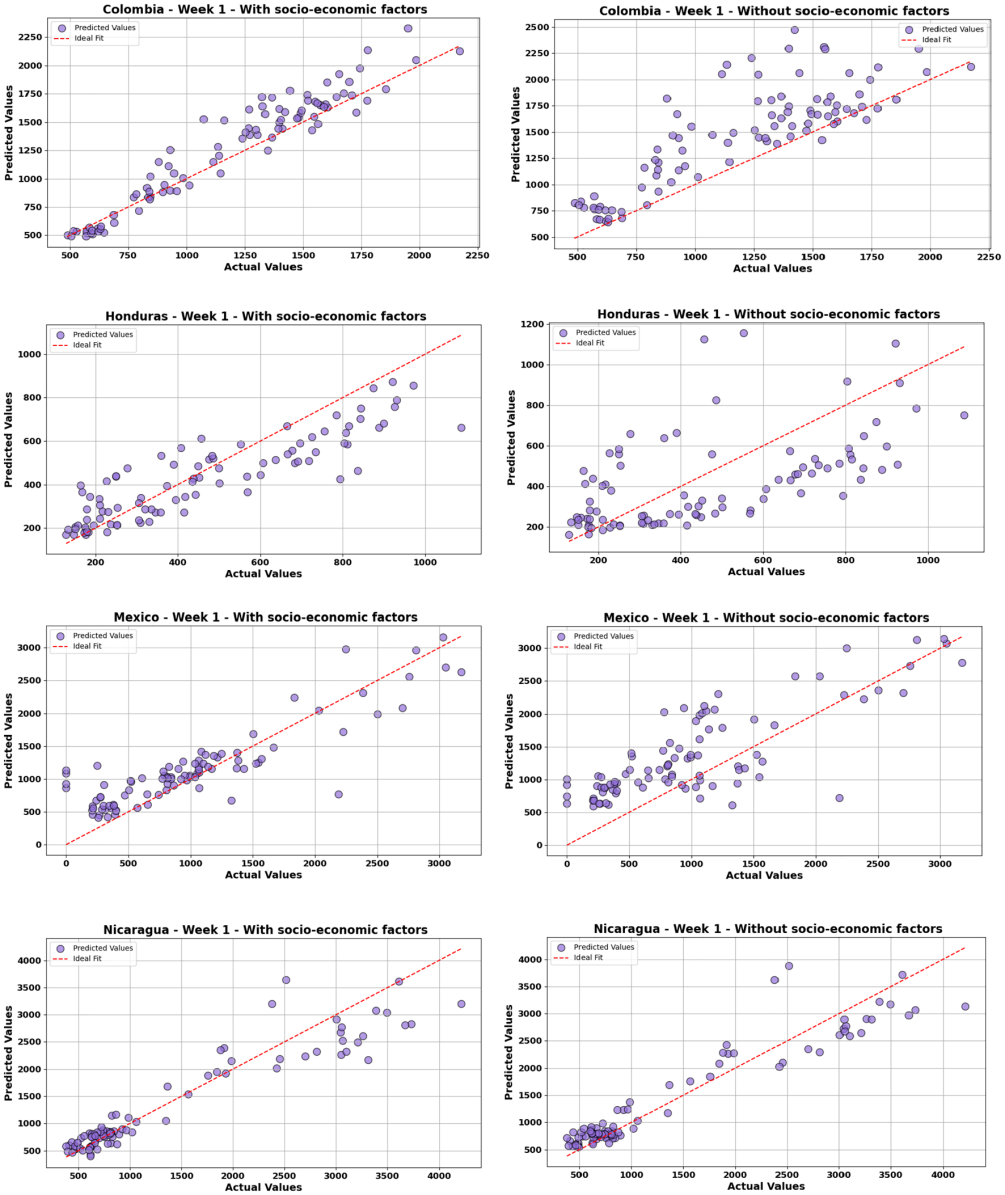
Comparison of STGCN model accuracy in dengue prediction with and without socio-economic factors. The left graph illustrates the model’s prediction accuracy when socio-economic factors are included in the training process. In contrast, the right graph displays the results obtained when these factors are excluded.

Figure 9 illustrates the scatter plot of the model’s predictions over a one-week forecast horizon under two distinct scenarios: one considering socio-economic factors in model training and the other excluding them. The horizontal axis denotes the actual values, while the vertical axis represents the values forecasted by the model. In this figure, the chart on the right corresponds to the model’s execution without socioeconomic factors, whereas the chart on the left includes these factors. To assess the accuracy of a model using a scatter plot, the closer the points are to the 45-degree diagonal line, the more accurately the predicted values match the actual values. Tight clustering of points around this line suggests high accuracy, while widely scattered points indicate lower accuracy. These plots are provided for exploratory visualization purposes. Our aim is to qualitatively illustrate how the inclusion of socio-economic factors may influence the distribution and stability of predictions. Based on the analysis of actual and predicted values, we observe substantial differences in the prediction of dengue cases with and without socio-economic factors. The data sets without socio-economic factors generally exhibit higher variability in predictions, showing significant fluctuations. In contrast, the inclusion of socio-economic factors yields more stabilized trends, indicating more accurate and reliable predictions of dengue cases. This discrepancy is especially pronounced for Colombia, Honduras, Peru, and Mexico. The highest R-squared improvement was observed in Honduras, with an increase of 136.36%, while Brazil showed the smallest improvement, with a modest increase of 1.03%. This highlights the crucial role that socio-economic factors play in the spread and prediction of dengue in these countries, offering more precise forecasts and potentially enabling better-targeted public health interventions. It is important to note that while the integration of socio-economic factors has generally enhanced predictive accuracy, there are instances, like those of Ecuador and Nicaragua, where this improvement is only marginal.

## Discussion

Our adapted STGCN framework achieved high predictive accuracy in forecasting dengue outbreaks, with $\hbox {R}^2$ values between 0.78 and 0.98 for different regions. This demonstrates the model’s ability to effectively capture complex temporal and spatial patterns. Based on the results reported in previous studies, our approach demonstrates prediction accuracy that is generally comparable to, and in certain instances surpasses, the established methods. For example, as highlighted in Table 3, our model’s performance shows consistency and occasional improvements across different scenarios, underscoring its reliability regardless of the methods or datasets employed. This means that the STGCN approach can be used as one of the accurate and robust models in predicting dengue especially in short-term forecasting scenarios. A noteworthy aspect is that most research and published articles do not specify whether the reported accuracy is achieved with or without data shuffling in time series. This distinction is crucial because, had we shuffled the data, our model’s accuracy could have been even higher. Based on^35,36^, maintaining the temporal order of observations between training and validation sets produces results that are more realistic in terms of both bias and variance compared to cross-validation methods. So, by preserving the sequential nature of the data, our model was able to capture the underlying temporal patterns, which is crucial for accurately predicting time-series events such as dengue outbreaks.

**Table 3:** Accuracy of some published studies on dengue prediction using different models. Note: In this table, the $\hbox {R}^2$ value is the maximum value reported in each study.

Authors	Algorithms	Region	Accuracy
Conde-Gutiérrez et al. 2024^38^	ANN	Mexico	$\hbox {R}^2$: 0.968
da Silva et al. 2024^39^	RF	Brazil	$\hbox {R}^2$: 0.949
Saleh et al. 2021^40^	LSTM	Malaysia	$\hbox {R}^2$: 0.750
da Silva et al, 2024^39^	RF	Colombia	$\hbox {R}^2$: 0.953
Francisco et al. 2024^41^	6 ML methods: RF, SVM, ANN	Philippines	$\hbox {R}^2$: 0.920
da Silva et al. 2024^39^	RF	Peru	$\hbox {R}^2$: 0.887
Silitonga et al. 2021^42^	ANN	Indonesia	$\hbox {R}^2$: 0.910

In a multi-country comparative evaluation, the STGCN model consistently outperformed the Random Forest algorithm, as evidenced by the relative RMSE, $\hbox {R}^2$, NSE and MASE values. Higher NSE values and lower MASE scores reflect improved correlation and reliability in time-series forecasting. This outcome highlights the framework’s effectiveness across diverse epidemiological settings and its enhanced ability to generalize in the presence of data variability. The findings support the model’s potential as a scalable tool for regional disease surveillance, especially in resource-variable contexts. Our findings are consistent with those reported by^18,22,24^, who explored the comparative effectiveness of advanced models versus traditional approaches in predicting dengue cases. Traditional models often struggled to achieve high accuracy due to the complexity of dengue transmission dynamics. While the present comparison was limited to RF, future work can extend benchmarking to epidemiologically established approaches such as SARIMA and the endemic–epidemic framework (hhh4). This will provide broader context for situating STGCN within the landscape of epidemic forecasting methods.

Also, according to our results, model performance varied notably across the assessed countries. The variation in model performance can be attributed to differences in data quality, transmission dynamics, and the spatio-temporal structure of dengue outbreaks. In Brazil, although the STGCN model outperformed RF, both models achieved their highest performance in this country across all metrics, suggesting that the availability of consistent and high-quality data enabled both models to learn effectively^37^. In contrast, regions such as Bolivia and Peru exhibited mixed results, where STGCN outperformed RF in capturing temporal variability (higher NSE), but RF showed slightly lower absolute errors (better MASE). These discrepancies highlight the importance of contextual factors, such as surveillance density, spatial connectivity, and outbreak regularity, in shaping model performance^37^. These findings underscore the epidemiological relevance of the model, as performance differences reflect surveillance quality, transmission heterogeneity, and contextual outbreak dynamics. Further investigation into the data and models may help explain variations in performance across countries and identify ways to improve the model’s effectiveness.

The success of our framework can be attributed to several factors. Firstly, the spatio-temporal structure of our deep learning model significantly contributed to the high prediction accuracy achieved. The STGCN model leverages graph convolutions to effectively capture spatial relationships within graph-structured data, enabling the identification of intricate patterns in the data. This dual consideration of space and time provided a comprehensive understanding of the factors influencing dengue transmission. Secondly, the algorithm’s ability to handle outliers effectively further improved its accuracy. Furthermore, the inclusion of a broader range of data sources, including climate data, socio-economic factors, and historical dengue cases, contributed to the enhanced predictive performance (see the subsection on Impact of socio-economic factors). While socio-economic variables improved predictive accuracy, the model should not be interpreted as identifying causal drivers of dengue risk without a dedicated interpretability framework. Based on these insights, the STGCN model enhances our understanding of the complex dynamics of disease transmission. Additionally, researchers can leverage the STGCN model to analyze intricate patterns in vector-borne diseases, such as malaria, Zika, or chikungunya, facilitating further investigations into the factors influencing vector-borne diseases transmission.

Despite the strengths of the STGCN model, several limitations warrant consideration, each pointing toward opportunities for future improvement. First, the model was optimized to minimize overall error, which may have inadvertently biased performance toward countries with higher dengue incidence. To address this, future research could explore weighted loss functions or stratified training approaches that balance performance across regions with varying disease burdens.

Second, training the model at the national level may mask regional climate heterogeneity, particularly in geographically diverse countries. Although the present study was conducted at the national scale as a methodological proof-of-concept across multiple regions, we acknowledge that this aggregation may limit the representation of local dengue transmission dynamics, and thus our results should not be interpreted at fine spatial scales. To more effectively capture local dynamics, future research may benefit from incorporating subnational data or adopting hierarchical modeling frameworks, thereby enabling more detailed epidemiological insights.

Third, while the use of a trainable adjacency matrix allowed the model to autonomously infer functional connectivity patterns from incidence, climatic, and socio-economic data, the absence of direct epidemiological inputs such as human mobility, migration, or vector distribution data may limit the biological interpretability of the learned graph. Accordingly, sensitivity analyses that compare trainable and fixed adjacency matrices could strengthen the biological plausibility of the inferred graph and provide an informative assessment of model robustness.

Lastly, while the model demonstrates strong performance for short-term forecasts, its reliability over longer horizons remains uncertain. Developing hybrid models or ensemble approaches may enhance long-term predictive capacity and support strategic planning.

Furthermore, our framework currently produces deterministic forecasts, which limits the statistical characterization of uncertainty. While we assessed stability through repeated runs and sensitivity to input scenarios, formal confidence intervals would require probabilistic forecasting approaches such as Bayesian inference, ensemble bootstrapping, or dropout-based uncertainty modeling. Incorporating such methods, together with standardized outbreak definitions, represents an important direction for future research to enhance interpretability, provide prediction intervals alongside point forecasts, and align with epidemiological reporting standards. In this context, probabilistic metrics would provide valuable evaluation tools, but they require probabilistic forecasts rather than deterministic case counts. We therefore highlight this as an important direction for future research.

## Conclusion

This study was conducted with the aim of applying and evaluating an alternative spatio-temporal method. In this research, we adapted a Spatio-Temporal Graph Convolutional Network (STGCN) model as a novel approach for forecasting dengue across nine countries in South and Central America. STGCN is a deep learning model initially developed for traffic forecasting. We chose the STGCN model for its strong potential in exploring spatio-temporal structures from the input to analyze intricate patterns. Additional benefits of the model include fast training, easy convergence, and a small number of parameters with flexibility and scalability. We tested our models using averaged data from nine countries in South and Central America.

The results demonstrate that STGCN consistently outperforms the Random Forest (RF) model in most scenarios, particularly in short-term forecasts (1–2 weeks), where it achieved higher accuracy and lower error rates. While both models showed declining performance over longer horizons, STGCN maintained greater temporal stability and adaptability, especially in countries with high data variability. By integrating environmental and socio-economic variables, factors often underrepresented in previous studies, our model aimed to improve predictive accuracy.

Overall, the efficiency and accuracy of the proposed deep learning model demonstrate its value in dengue prediction research. By combining spatio-temporal data with advanced convolutional neural networks, our approach highlights the growing importance of deep learning in addressing global health challenges. Despite certain limitations, such as uneven data quality across countries and reduced accuracy in long-term forecasts, the STGCN model proved to be a robust, scalable, and adaptable tool for epidemic prediction. Its ability to generalize across diverse epidemiological settings suggests strong potential for public health applications, including early warning systems and targeted interventions for vector-borne diseases.

To better understand how effective the model truly is, future research could involve training it on data from different regions and testing its performance across various timeframes and spatial scales. It would also be worthwhile to explore hybrid modeling techniques, use higher-resolution geographic data, and extend the forecast period to improve accuracy. Additionally, applying this approach to other vector-borne diseases like Zika or chikungunya could reveal how broadly useful spatio-temporal deep learning models might be for global health monitoring and epidemic prediction.

Beyond the demonstrated effectiveness of STGCN in short-term dengue forecasting (up to one month), our future work will focus on extending the forecasting horizon to evaluate long-term predictive performance under different scenario-based settings. While the current results already show promising accuracy, further exploration is needed to assess the model’s robustness over longer horizons. Comparative studies with other advanced spatio-temporal deep learning models such as Temporal Graph Attention Networks (TGAT), Graph WaveNet, and Transformer-based spatio-temporal architectures would provide valuable insights. These comparisons will help situate STGCN within the broader landscape of epidemic forecasting methods and highlight its strengths and limitations across varying temporal and spatial contexts.

## Supplementary Information

Supplementary Information.

## Data Availability

The dataset on Dengue disease is available at Open Dengue Database (https://opendengue.org). Weather and climate factors are available at NASA data source (https://science.nasa.gov/earth/data/climate-data). The socio-economic factors and environmental factors are available at the World Bank Database (https://data.worldbank.org/indicator).

## Code availability

The original implementation of the Spatio-Temporal Graph Convolutional Networks (ST-GCN) used in this study is publicly available on GitHub. The code can be accessed at: https://github.com/VeritasYin/STGCN_IJCAI-18. The implementation of Random Forest Regression used in this study is available through the scikit-learn library in Python. The official documentation and source code can be accessed at: https://scikit-learn.org.
