# Supplementary material for: Innovative application of a traffic-prediction spatio-temporal graph convolutional network for dengue disease forecasting
Source: Sci Rep. 2026 Jan 17;16:2594. doi: 10.1038/s41598-026-36225-7 (PMC12820264; doi:10.1038/s41598-026-36225-7)
Supplement: Supplementary file 1 — Supplementary Information. [file 41598_2026_36225_MOESM1_ESM.pdf]

Supplementary Material

Table S1 Sensitivity analysis of model performance with and without data augmentation, reported as R^2 and relative RMSE across nine countries. Higher R^2 and lower RMSE values indicate superior predictive performance.

**Country**	**Model Setting**	$\mathbf{R^2}$	**Rel. RMSE**
Brazil	With interpolation	0.98	0.196
	Without interpolation	0.98	0.199
Colombia	With interpolation	0.93	0.142
	Without interpolation	0.90	0.177
Bolivia	With interpolation	0.91	0.303
	Without interpolation	0.25	1.043
Peru	With interpolation	0.93	0.184
	Without interpolation	0.79	0.489
Ecuador	With interpolation	0.83	0.243
	Without interpolation	0.78	0.285
Nicaragua	With interpolation	0.90	0.271
	Without interpolation	0.85	0.418
El Salvador	With interpolation	0.83	0.432
	Without interpolation	0.36	0.693
Honduras	With interpolation	0.78	0.309
	Without interpolation	0.40	0.472
Mexico	With interpolation	0.78	0.422
	Without interpolation	0.76	0.487
